# Ten novel insertion/deletion variants in *MECP2* identified in Japanese patients with Rett syndrome

**DOI:** 10.1038/s41439-019-0078-2

**Published:** 2019-10-18

**Authors:** Eri Takeshita, Aritoshi Iida, Chihiro Abe-Hatano, Eiji Nakagawa, Masayuki Sasaki, Ken Inoue, Yu-ichi Goto

**Affiliations:** 1 Department of Child Neurology, National Center Hospital, National Center of Neurology and Psychiatry (NCNP), Kodaira, Tokyo, 187-8551 Japan; 2 Department of Clinical Genome Analysis, Medical Genome Center, NCNP, Kodaira, Tokyo, 187-8551 Japan; 3 Department of Mental Retardation and Birth Defect Research, National Institute of Neurology, NCNP, Kodaira, Tokyo, 187-8551 Japan; 4 Medical Genome Center, NCNP, Kodaira, Tokyo, 187-8551 Japan

**Keywords:** DNA sequencing, Genetics research

## Abstract

Rett syndrome (RTT) is an X-linked progressive and severe neurological disorder caused by mutations in the gene encoding methyl CpG binding protein 2 (*MECP2*). Among the 49 typical RTT patients examined, we identified 10 novel and eight known insertion/deletion variants, and 31 known pathogenic variants in *MECP2*. The pathogenic variants presented here should be a useful resource for examining the correlation between the genotypes and phenotypes of RTT.

Rett syndrome (RTT) is a progressive neurodevelopmental disorder that affects brain development and function in females, with a prevalence of one in 10,000 worldwide^1^. Typical RTT is caused by mutations in the gene encoding methyl-CpG binding protein 2 (*MECP2*)^2^. A database of a large collection of *MECP2* variants was established in 2002 (RettBASE: http://mecp2.chw.edu.au/index.shtml)^3^. To date, associations between clinical phenotypes and related genetic variants for *MECP2* as well as other RTT-associated genes, including *CDKL5* and *FOXG1*, are available.

Here, we report a total of 49 RTT patients with 10 novel insertion/deletion variants, eight known insertion/deletion variants and 31 known pathological variants.

All patients were diagnosed with typical RTT by Japanese child neurology experts according to the international diagnostic criteria for RTT. Clinical information and samples from the patient and parents were obtained with written informed consent. The study was approved by the ethical committee of NCNP. Genomic DNA was extracted from peripheral blood using a standard protocol. We first used the MLPA method (MRC-Holland, DL Amsterdam, The Netherlands) to identify the structural abnormalities in the *MECP2* locus. In the patients excluded for structural abnormalities, we amplified all coding exons of *MECP2* and their exon-intron boundaries by PCR and directly sequenced the PCR products using the Applied Biosystems 3730 DNA analyzer (Thermo Fisher, USA).

The insertion/deletion mutations were detected in 18 (36.7%) of 49 patients with RTT (Table 1). Among the 18 patients, 10 (55.6%) were considered novel by a comparison of our data with the known insertions/deletions deposited in the public databases, including RettBASE, gnomAD, Human Genome Mutation Database Professional 2019.2 and ClinVar. Representative data of pedigrees and sequences of the recombination breakpoints from three families are shown in Fig. 1. Patient 470 showed the insertion/deletion variant at c.1158_1258delinsCCGAGGGTGGCTCC. Patient 488 showed the deletion at c.1168_*539del. Patient 587 showed the insertion/deletion at c.1367_*791delinsCGC. Five (Patients 187, 470, 488, 559, and 587) had lost only exon 4. In addition, Patient 269 has a complex rearrangement with a 2609 bp deletion, including exon 3 and flanking introns, accompanied by two nucleotide substitution and a 25 bp deletion in exon4, c. [27-1707_c.378-206del; 1159_1160CC>AG; 1164_1188del], occurred in *cis*. The other five showed intragenic deletion involving exon 4 in the *MECP2* locus. Hardwick et al. (2007) reported that 12 out of 21 patients (57%)^4^ experienced breakpoints within the “deletion-prone region (DPR)”, which is characterized by short direct repeat elements and is also known as the hotspot for the smaller deletions^5,6^. In this study, the breakpoints in seven novel insertion/deletion variants (7/10: 70%) were located within the DPR. In the breakpoints of Patients 269, 289 and 451, no repetitive sequences were found adjacent to the breakpoint. These findings suggest that the de novo deletion events involving *MECP2* can be unique to families and that homology-mediated mechanisms are unlikely to be associated with these events.

**Table 1:** Insertion/deletion variants in *MECP2* in Japanese patients with RTT

Family ID	Sex	Age (year:month)	Substitution		Exon/Intron deleted	DPR (1057–1209)	Deleted domain	Note
			Nucleotide	Amino acid				
187	F	3 years 2 months	c.816_819del	p.(Gly273Valfs*)	Exon 4		TRD	This study
269	F	3 years	c.[27–1707_c.378-206del; 1159_1160CC > AG; 1164_1188del]	p.[Pro387Ser; Pro388fs*]	Intron 2-Exon 4	Deleted	CTD	
289	F	2 years 11 months	c.378-375_c.1193del		Intron 3-Exon 4	Deleted	MBD, ID, TRD	
451	F	3 years	c.27–7899_c.1137del		Intron 2-Exon 4	Deleted	NTD, MBD, ID, TRD	
470	F	27 years 1 month	c.1158_1258delinsCCGAGGGTGGCTCC	p.(Pro387_Pro419delinsArgGlyTrpLeu)	Exon 4	Deleted	CTD	
487	F	36 years 11 months	c.27-?_378 + ?del	?	Intron 2-Exon 4		?	
488	F	2 years 4 months	c.1168_*539del	p.(Pro390_Ser486delinsValArgSerHisProTrpTrpLeuLysSerGlyProThrProAlaProIleGlnAsnTrpGlnGlyArgPheThrGlyGlnGluSerGlyThrCysLeuLeuGlnLeuTrpHisGly)	Exon 4	Deleted	CTD	
500	F	2 years 1 month	c.27-?_378 + ?del	?	Intron 2-Exon 4		?	
559	F	2 years 1 month	c.1038_1195delinsAGCA	p.(Ser346Argfs*)	Exon 4	Deleted	CTD	
587	F	10 years 2 months	c.1367_*791delinsCGC	p.(Gly456_Ser486delinsAlaLeuGlyGlnGlyAlaGlyArgLeuAlaTrpGlyGlnAlaGlyGlnSerThrAlaGly)	Exon 4	Deleted	CTD	
288	F	2 years 6 months	c.806del	p.(Gly269Alafs*)	Exon 4		TRD	Wan M et al. (1999)
376	F	4 years 2 months	c.47–57del	p.(Gly16Glufs*)	Exon 4		NTD	Mnatzakanian GN et al. (2004)
467	F	2 years 1 month	c.696del	p.(Lys233Argfs*)	Exon 4		TRD	Obata K et al. (2000)
497	F	1 year 1 month	c.710dupG	p.(Gly238Trpfs*)	Exon 4		TRD	Hoffbuhr K et al. (2001)
511	F	4 years	c.808del	p.(Arg270Glufs*)	Exon 4		TRD	Obata K et al. (2000)
539	F	5 years 6 months	c.1157_1200del	p.(Leu386fs*)	Exon 4	Deleted	CTD	RettBASE
555	F	3 years 2 months	c.1154_1197del	p.(Pro385Hisfs*)	Exon 4	Deleted	CTD	Bienvenu T et al. (2002)
572	F	1 year 9 months	c.710del	p.(Gly237fs*)	Exon 4		TRD	Amir RE et al. (2000)

NM_004992.3(MECP2_i001)

*ID* Interdomain, *CTD* C-terminal domain, *MBD* methyl CpG binding domain, *NTD* N-terminal domain, *TRD* transcriptional repression domain

**Fig. 1:**
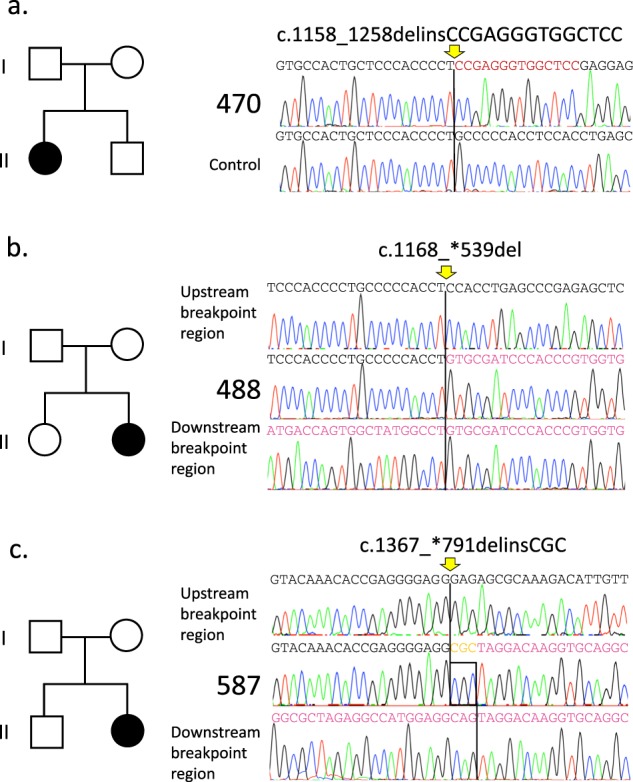
Sequence analysis of the breakpoints of *MECP2* structural variants in three RTT patients. **a**–**c** Pedigrees of each family (left). Nucleotide sequences of *MECP2* in patient 470, 488, 587, and controls spanning the breakpoint of each structural variant. All samples were amplified by PCR and then cloned into a plasmid vector, followed by direct sequencing of the junction fragments

In addition, we identified known pathogenic variants in 31 patients (Supplementary Table 1). No novel change was identified, suggesting that the molecular basis for recurrent de novo nucleotide substitutions in *MECP2* is common among the different populations.

The list of *MECP2* variants found in 49 Japanese patients with RTT should provide a useful resource to further examine the correlation between genotypes and disease phenotypes.

## Supplementary information

Supplementary Table 1

Supplementary References

## Data Availability

The relevant data from this Data Report are hosted at the Human Genome Variation Database at 10.6084/m9.figshare.hgv.2648 10.6084/m9.figshare.hgv.2651 10.6084/m9.figshare.hgv.2654 10.6084/m9.figshare.hgv.2657 10.6084/m9.figshare.hgv.2660 10.6084/m9.figshare.hgv.2663 10.6084/m9.figshare.hgv.2666 10.6084/m9.figshare.hgv.2669 10.6084/m9.figshare.hgv.2672 10.6084/m9.figshare.hgv.2675 10.6084/m9.figshare.hgv.2678 10.6084/m9.figshare.hgv.2681 10.6084/m9.figshare.hgv.2684 10.6084/m9.figshare.hgv.2687 10.6084/m9.figshare.hgv.2690 10.6084/m9.figshare.hgv.2693 10.6084/m9.figshare.hgv.2696 10.6084/m9.figshare.hgv.2699 10.6084/m9.figshare.hgv.2702 10.6084/m9.figshare.hgv.2705 10.6084/m9.figshare.hgv.2708 10.6084/m9.figshare.hgv.2711 10.6084/m9.figshare.hgv.2714 10.6084/m9.figshare.hgv.2717 10.6084/m9.figshare.hgv.2720 10.6084/m9.figshare.hgv.2723 10.6084/m9.figshare.hgv.2726 10.6084/m9.figshare.hgv.2729 10.6084/m9.figshare.hgv.2732 10.6084/m9.figshare.hgv.2735 10.6084/m9.figshare.hgv.2738 10.6084/m9.figshare.hgv.2741 10.6084/m9.figshare.hgv.2744 10.6084/m9.figshare.hgv.2747 10.6084/m9.figshare.hgv.2750 10.6084/m9.figshare.hgv.2753 10.6084/m9.figshare.hgv.2756 10.6084/m9.figshare.hgv.2759 10.6084/m9.figshare.hgv.2762 10.6084/m9.figshare.hgv.2765 10.6084/m9.figshare.hgv.2768 10.6084/m9.figshare.hgv.2771 10.6084/m9.figshare.hgv.2774 10.6084/m9.figshare.hgv.2777 10.6084/m9.figshare.hgv.2780 10.6084/m9.figshare.hgv.2783
