# Supplementary material for: Ten novel insertion/deletion variants in MECP2 identified in Japanese patients with Rett syndrome
Source: Hum Genome Var. 2019 Oct 18;6:48. doi: 10.1038/s41439-019-0078-2 (PMC6804785; doi:10.1038/s41439-019-0078-2)
Supplement: Supplementary file 2 — Supplementary References [file 41439_2019_78_MOESM2_ESM.docx]

**Ten novel insertion/deletion variants in *MECP2* identified in Japanese patients with Rett syndrome**

Eri Takeshita^1^, Aritoshi Iida^2^, Chihiro Abe-Hadano3, Eiji Nakagawa1, Ken Inoue^3^ and Yu-ichi Goto^3, 4^

**References for Table 1**

Amir RE, Van den Veyver IB, Schultz R, Malicki DM, Tran CQ, Dahle EJ, et al. Influence of mutation type and X chromosome inactivation on Rett syndrome phenotypes. Ann Neurol. **47**,670-679 (2000).

Bienvenu T, Villard L, De Roux N, Bourdon V, Fontes M, Beldjord C, et al. Spectrum of MECP2 mutations in Rett syndrome. Genet Test. **6**, 1-6 (2002).

Hoffbuhr K, Devaney JM, LaFleur B, Sirianni N, Scacheri C, Giron J, et al. MeCP2 mutations in children with and without the phenotype of Rett syndrome.

Neurology. **56**, 1486-1495 (2001).

Mnatzakanian GN, Lohi H, Munteanu I, Alfred SE, Yamada T, MacLeod PJ, et al. A previously unidentified MECP2 open reading frame defines a new protein isoform relevant to Rett syndrome. Nat Genet. **36**, 339-341 (2004).

Obata K, Matsuishi T, Yamashita Y, Fukuda T, Kuwajima K, Horiuchi I, et al. Mutation analysis of the methyl-CpG binding protein 2 gene (MECP2) in patients with Rett syndrome. J Med Genet. **37**, 608-610 (2000).

Wan M, Lee SS, Zhang X, Houwink-Manville I, Song HR, Amir RE, et al. Rett syndrome and beyond: recurrent spontaneous and familial MECP2 mutations at CpG hotspots. Am J Hum Genet. **65**, 1520-1529 (1999).

**References for Supplementary Table 1**

Amir RE1, Van den Veyver IB, Wan M, Tran CQ, Francke U, Zoghbi HY. et al. Rett syndrome is caused by mutations in X-linked MECP2, encoding methyl-CpG-binding protein 2. Nat Genet. **23**, 185-188 (1999).

Bienvenu T, Villard L, De Roux N, Bourdon V, Fontes M, Beldjord C, et al. Spectrum of MECP2 mutations in Rett syndrome. Genet Test. **6**, 1-6 (2002).

Campos M Jr, Abdalla CB, Santos-Rebouças CB, dos Santos AV, Pestana CP, Domingues ML, et al. Low significance of MECP2 mutations as a cause of mental retardation in Brazilian males. Brain Dev. **29**, 293-297 (2007).

Cheadle JP, Gill H, Fleming N, Maynard J, Kerr A, Leonard H, et al. Long-read sequence analysis of the MECP2 gene in Rett syndrome patients: correlation of disease severity with mutation type and location. Hum Mol Genet. **9**, 1119-1129 (2000).

De Bona C, Zappella M, Hayek G, Meloni I, Vitelli F, Bruttini M, et al. Preserved speech variant is allelic of classic Rett syndrome. Eur J Hum Genet. **8**, 325-330 (2000).

Obata K, Matsuishi T, Yamashita Y, Fukuda T, Kuwajima K, Horiuchi I, et al. Mutation analysis of the methyl-CpG binding protein 2 gene (MECP2) in patients with Rett syndrome. J Med Genet. **37**, 608-610 (2000).

Wan M, Lee SS, Zhang X, Houwink-Manville I, Song HR, Amir RE, et al. Rett syndrome and beyond: recurrent spontaneous and familial MECP2 mutations at CpG hotspots. Am J Hum Genet. **65**, 1520-1529 (19999).
